# Prediction of leukemia peptides using convolutional neural network and protein compositions

**DOI:** 10.1186/s12885-024-12609-8

**Published:** 2024-07-26

**Authors:** Seher Ansar Khawaja, Muhammad Shoaib Farooq, Kashif Ishaq, Najah Alsubaie, Hanen Karamti, Elizabeth Caro Montero, Eduardo Silva Alvarado, Imran Ashraf

**Affiliations:** 1 School of System and Technology, University of Management and Technology, Lahore, 54000 Pakistan; 2 Department of Computer Sciences, College of Computer and Information Sciences, Princess Nourah bint Abdulrahman University, P.O.Box 84428, Riyadh, 11671 Saudi Arabia; 3 Universidad Europea del Atlántico, Isabel Torres 21, 39011 Santander, Spain; 4 Universidad Internacional Iberoamericana Arecibo, Puerto Rico, 00613 USA; 5 Universidade Internacional do Cuanza, Cuito, Bié Angola; 6 Universidad Internacional Iberoamericana, Campeche, 24560 México; 7 Universidad de La Romana, La Romana, República Dominicana; 8 Information and Communication Engineering, Yeungnam University, Gyeongsan, 38541 Korea

**Keywords:** Leukemia detection, Protein sequences, Deep learning, Convolutional neural network

## Abstract

Leukemia is a type of blood cell cancer that is in the bone marrow’s blood-forming cells. Two types of Leukemia are acute and chronic; acute enhances fast and chronic growth gradually which are further classified into lymphocytic and myeloid leukemias. This work evaluates a unique deep convolutional neural network (CNN) classifier that improves identification precision by carefully examining concatenated peptide patterns. The study uses leukemia protein expression for experiments supporting two different techniques including independence and applied cross-validation. In addition to CNN, multilayer perceptron (MLP), gated recurrent unit (GRU), and recurrent neural network (RNN) are applied. The experimental results show that the CNN model surpasses competitors with its outstanding predictability in independent and cross-validation testing applied on different features extracted from protein expressions such as amino acid composition (AAC) with a group of AAC (GAAC), tripeptide composition (TPC) with a group of TPC (GTPC), and dipeptide composition (DPC) for calculating its accuracies with their receiver operating characteristic (ROC) curve. In independence testing, a feature expression of AAC and a group of GAAC are applied using MLP and CNN modules, and ROC curves are achieved with overall 100% accuracy for the detection of protein patterns. In cross-validation testing, a feature expression on a group of AAC and GAAC patterns achieved 98.33% accuracy which is the highest for the CNN module. Furthermore, ROC curves show a 0.965% extraordinary result for the GRU module. The findings show that the CNN model is excellent at figuring out leukemia illnesses from protein expressions with higher accuracy.

## Introduction

Leukemia is a form of melanoma damaging blood cells and bone marrow that has various types of cancer diseases. The most common types of Leukemia disease include polycythemia vera, chronic lymphocytic leukemia, myelodysplastic syndrome, and acute lymphoblastic leukemia [1]. Leukemia impacts the development of genes and proteomics that develop TCF3-HLF-positive acute lymphoblastic leukemia in the body [1]. Leukemia affects mostly adults over the age of 55, but it can also occur in children under 15 years. In Pakistan, human T-cell leukemia affects approximately 80% of children, and only 9% of them are treated but 71% of them are left untreated which leads to deaths [1, 2]. The symptoms of leukemia, a kind of blood cancer, include exhaustion, various infections, and brain hemorrhage or blood clotting. Leukemia interacts with the ability of the human body to manufacture healthy blood cells and antibodies. It may significantly affect a person’s antibodies and psychological health [2, 3]. We are able to analyze structured and unstructured data, including clinical notes, test results, diagnoses, and prescription information, utilizing medical record data by implementing deep learning models that provide the highest degree of efficiency and accuracy [4, 5].

Deep learning applications in biotechnology are rapidly growing to anticipate leukemia disease by taking protein expressions for experiments [4–7]. The implementation of deep learning algorithms can predict protein expression interactions in leukemia disorders [8]. Therefore, many medical sectors that depend on these proteins to improve the prediction accuracy of leukemia illnesses through protein expression relationships, would be greatly impacted by an efficient deep-learning approach. The basic purpose of using multiple modules of deep learning is to predict the leukemia disease at the first stage [5, 8]. In deep learning, methodologies like sequence alignment and computer modeling give ideal accuracy and computational effectiveness for the prediction of leukemia disease [6, 7].

Blood cancers, which affect white blood cells, organs, and bone marrow, exhibit unique pathologies. Unlike other cancers that give rise to solid tumors, leukemia manifests by producing an abnormally high number of white blood cells, disrupting normal vascular functions. Machine learning methodologies play a substantial role in leukemia treatment, be it for identifying different myeloma types or detecting the disease in individuals. However, this severe form of cancer presents significant medical challenges, often requiring specialized doctors and pathologists to manually examine blood samples under a microscope for diagnosis. In managing cancer cases, practitioners in this field can greatly benefit from tools like image processing and pattern recognition.

Deep learning models have shown their potential in accurate disease diagnosis, compared to machine learning models [9–11]. In this study, various deep-learning models are employed using various protein compositions for leukemia classification. This research provides deep learning-established results that outperform other approaches in reliably categorizing sequences of proteins associated with acute lymphocytic leukemia (ALL). Besides experimenting with different protein compositions, cross-validation is also carried out for in-depth evaluation of models’ robustness and generalization.

This research presents a state-of-the-art analysis of growing leukemia disease in protein by using multiple deep-learning approaches. By applying the convolutional neural network (CNN), recurrent neural network (RNN), multilayer perceptron (MLP), and gated recurrent unit (GRU) models, this study gives an accurate prediction of leukemia disease in bone marrow and blood cell tissues for detection and diagnosis of cancer at first stage. Experimental findings reveal that deep learning models can be a potential solution for the automated detection of leukemia disease. A diverse range of deep learning approaches such as CNN, RNN, MLP, and GRU are adopted for their efficacy in preconditioning, segmentation, feature extraction, and classification. It is vital to highlight that feature extraction necessitates a high level of skill, as bad segmentation can degrade feature selection and, as a result, classification accuracy.

This research presents a detailed analysis of the growing importance of leukemia genes in protein sequences and their significant involvement in leukemia disease. By implementing multiple deep learning techniques such as sequence alignment and computer modeling, the accuracy and computational efficiency of anticipating leukemia diseases in the expression of protein interactions improved. Precision prediction of leukemia diseases in the interactions between blood malignant tissues and bone marrow has been obtained by implementing advanced deep learning models, resulting in enhanced cancer detection and diagnosis. The following highlights the contributions of this study

- A detailed analysis of leukemia genes in protein sequences and their impact on leukemia disease is presented.
- A comparative analysis and feasibility of various deep learning models is investigated within the context of acute lymphocytic leukemia which can be beneficial for building advanced and more accurate computer-aided diagnosis systems.
- The importance of various features and their impact on the detection accuracy of deep learning models is also investigated. In this regard, amino acid composition, dipeptide composition, tripeptide composition, composition/transition/distribution, group of tripeptide composition, and group of amino acid composition are utilized to evaluate the performance of the models.
- Datasets were collected and processed for processing. The Swiss/Uniprot database web server was utilized to collect leukemia protein sequences and the data was filtered for identical and similar sequences. In addition, CD-HIt configuration was used to remove redundant samples for improved accuracy.
- Performance evaluation of models is carried out concerning the independence testing, accuracy, F1 score, receiver operating curve, and other evaluation parameters.

The remainder of this study is structured as follows. Related work section introduces the work about disease detection for cancerous types, and materials and methods are presented in Materials and methods section. Results and discussion section is about the results of prediction of leukemia peptides using deep learning models. In the end, Conclusion section gives the conclusion.

## Related work

The complexity of bioinformatics and biomedical data presents methodological challenges when applying machine learning approaches to extract features, classification, and visualize data. To address these challenges, the study [12] proposes the utilization of clustering to identify predictive subgroups in cases of leukemia and peptide diseases. In the study, two tests were conducted to condense features into binary vectors using k-means clustering and ten different distance metrics. The authors employed multidimensional scaling to illustrate the condensed feature vectors. Using the Kaplan-Meier estimates technique and the Cox proportional hazard model, survival analysis was carried out to investigate the predictive benefits. The detected clusters and survival outcomes were shown to be statistically significantly correlated by the researchers. Notably, significant associations were reported between overall survival (*P* = 0.0164) and the time elapsed between diagnosis and therapy (*P* = 0.0039). Through the use of multidimensional scaling, the clusters were effectively distinguished, revealing a gradient that corresponded to a pattern of extended survival. Individuals with prolonged continuity exhibited mutations in the immunoglobulin heavy-chain variable region gene glycoprotein (IGHV), a lack of the Zap 70 pattern, a predominance of females, and a younger age.

Accurate diagnosis of diseases, such as cancer, is essential in biomedical procedures, where gene products are employed to detect proteins based on gene expression levels. However, the extensive dimensions of gene expression data render them impractical for analysis using conventional statistical methods. The study [13] aims to identify leukemia peptides using innovative techniques. A dataset comprising 22,283 proteins from the Gene Transcription Collection repository’s leukemia proteomic data underwent preprocessing, involving Python’s normalization tests and principal component analysis, before the application of deep neural networks. The results indicated that deep learning surpassed traditional methods, achieving accuracy rates of 63.33% and 96.67% for deep neural networks with three hidden layers and a single-layer neural network, respectively. The utilization of modern techniques, such as deep learning, has the potential to enhance disease accuracy and performance, and it should be implemented in cancer detection and the immunogenic identification of various tumor types.

In the field of bioinformatics, the use of machine learning has become increasingly common for cancer prediction. However, deep learning, which is relatively new, has sparked debates regarding its effectiveness. Only a limited number of studies have explicitly compared deep neural networks with traditional machine learning approaches, and the results have varied. The study [14] conducts a comprehensive assessment of deep learning’s performance in cancer prediction across 22 protein expression computation tasks. The study investigates critical input factors and compares neural networks to established standard procedures. One specific task involves predicting the presence of cancer; however, the class distribution is significantly imbalanced, with 92.7% of samples categorized as cancer. Additionally, the study assesses the effectiveness of various transfer methods through several experiments and scenarios.

In [15], researchers shared valuable insights with their peers by reviewing various image-processing methods used in machine learning for leukemia diagnosis. Leukemia, a form of blood cancer, is characterized by the production of abnormal white blood cells in the bone marrow. It is categorized into two main types: acute leukemia, which progresses rapidly, and chronic leukemia, which develops more slowly. Each type can be further subdivided into two subtypes lymphocytic and myeloid. Additionally, this review examined the advantages and disadvantages of relevant research in this field.

CNN approaches have shown remarkable performance in effectively categorizing cancerous leukocytes. Additionally, computer-aided diagnostic (CAD) models prove highly effective in detecting leukemia and assisting clinicians in early disease detection. The objective of [16] is to develop a deep learning model exclusively for the classification of leukemic B-lymphoblasts. Data augmentation techniques were employed to handle limited datasets and build reliable and accurate deep-learning methodologies. Transfer learning was used to expedite learning and enhance the proposed network’s performance. The CNN model successfully harmonizes attributes extracted through systematically designed deep learning techniques, achieving a test accuracy of 100% for both cancerous and healthy cases, as well as for the ResNet-34 and DenseNet-121 models. These models also demonstrated perfect statistics and F1 scores. For acute myeloid leukemia (AML) prediction, the accuracy reached 99.66%, with precision at 1.0%, recall at 0.99%, and an F1 score of 0.98%. ResNet-34 exhibited a precision, recall, and F1 score of 99.74% for chronic lymphocytic leukemia (CLL), while this class achieved a precision, recall, and F1 score of 0.99%. For chronic myelogenous leukemia (CML) prediction, ResNet-34 achieved an accuracy of 99.73%, a precision of 0.99%, a recall of 1.0%, and an F1 score of 0.98.

AML, a form of blood malignancy with multiple subtypes, exhibits significant associations between specific recurrent chromosomal abnormalities and the response to therapy, duration of remission, and overall survival. Consequently, these abnormalities are utilized to categorize patients into three risk groups: favorable, intermediate, and adverse. However, the relationship between gene expression and these risk categories remains unclear. The gene expression patterns closely resembled those of these risk groups, suggesting that they could offer valuable insights into the origin of lymphoma. In [17], the authors propose using Bayesian ordinal response models to identify risk groups based on homological physiognomic information. To model the multidimensional series of responses and expected risk groups, we employ a range of prior distributions, including spike-and-slab average, spike-and-slab extensible acceleration, and regression-based strategies with factor integration indicators. The authors employ hypothesis tests with the Bayes factor as a metric for identifying relevant genes.

ALL, a type of cancer characterized by excessive lymph node proliferation in red blood cells, requires cost-effective and time-efficient diagnostic screening methods. Initial screening using peripheral blood smear (PBS) images is crucial due to its ease of use. However, challenges arise from symptomatic errors, non-specific prodromes, and the wide range of ALL symptoms that can impact the examination process. To address these challenges, the study [18] focuses on the utilization of machine-learning classifiers in conjunction with the Grey Wolf optimization algorithm for feature selection, differentiating between benign and malignant acute lymphoblastic leukemia. An adaptive threshold technique is used to enhance contrast and reduce defects in the photographs. The model is based on the Grey Wolf optimized operation technique, specifically designed for feature reduction. The combined classifier categorizes myeloma into both cancerous and benign types. After applying the Grey Wolf refinement approach, the model achieves 99.69% accuracy, 99.5% sensitivity, and 99% specificity. A comparative analysis of alternative classification algorithms has been conducted to validate the proposed framework. The discussed studies provide significant insights into the current state of leukemia classification and a brief summary of such approaches is given in Table 1.

**Table 1:** Overview and comparison of discussed works

Ref.	Architecture	Accuracy	F1 score	Recall	Precision	MCC
[12]	Cox proportional hazard model	(P 14.0039 for generalization surviving; P 14.0064 for moments from determining to treatment.)				
[13]	DL (single-layer neural network and DNNs)	63.33 and 96.67	-	-	-	-
[14]	DL used in MLP	unbalanced 92.7%	-	-	-	-
[15]	DL	97% (Reviewed)	-	-	-	-
[16]	ResNet-34, DenseNet- 121’s	100%	100%	100%	100%	-
[17]	Bayesian variable model	$\pi$ j- Beta(0.01,0.19) is 6.58, 6.53, 3.54 for Model 11, Model ll, and model iv				
[18]	ML multiple classifiers (RF, NB, SVM, KNN) RF Is Best to Perform Result	99.69%	99.5%	99%	99%	-

## Materials and methods

**Fig. 1:**
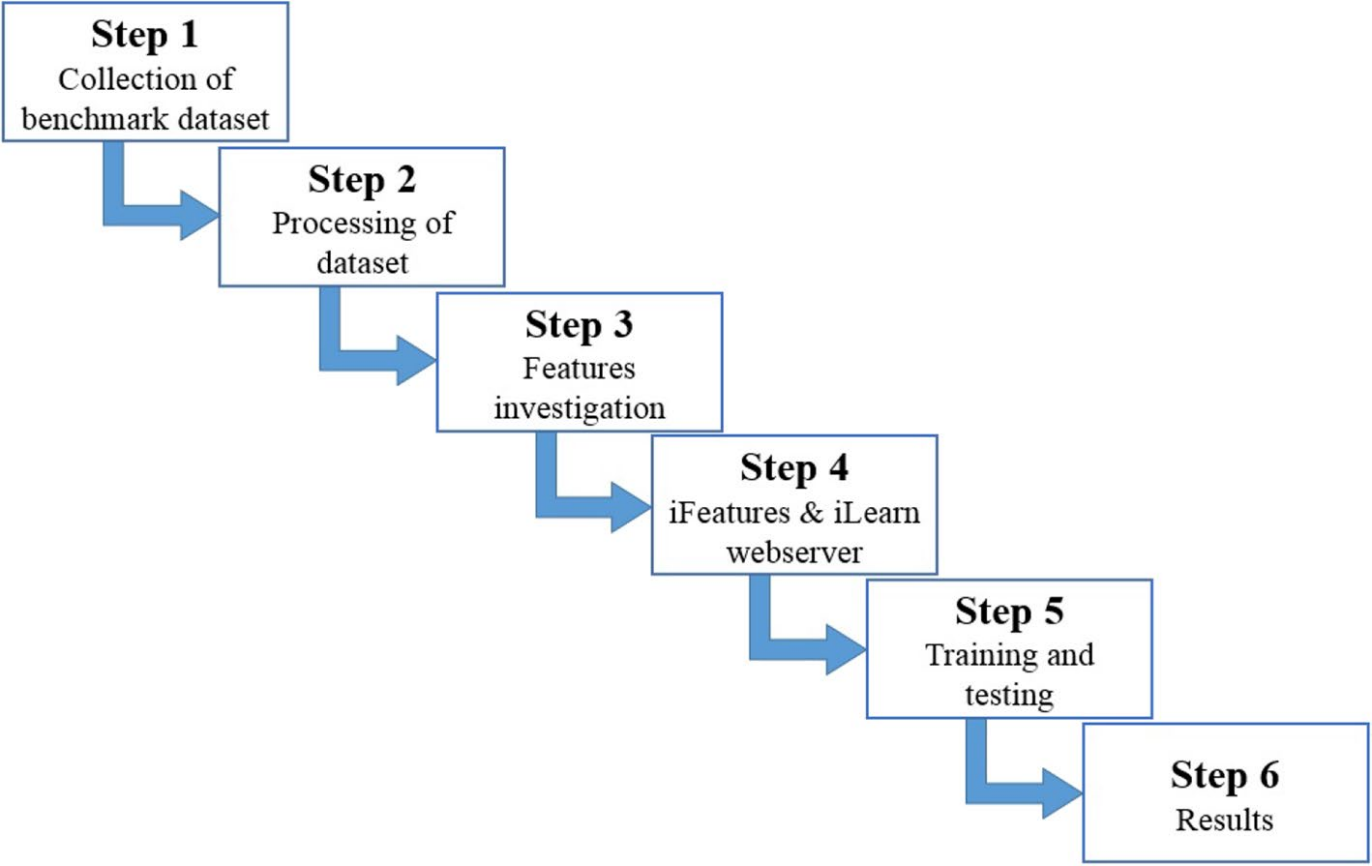
Layer-wise flow of the applied methodology

This section is about the adopted methodology, the dataset used for experiments, and the deep learning models employed in this study. Figure 1 shows the workflow of the adopted methodology.

### Collection of datasets

Datasets were used to create training and test datasets for deep learning models, comprising both positive and negative peptide samples. The anti-inflammatory analysis [8], and anti-cancer [19] datasets were collected from published papers, and the leukemia dataset was retrieved from the UniProt/Swiss-Prot protein web server database (www.uniprot.org) and filtered for identical and similar sequences. The leukemia protein sequences were extracted from the UniProt/Swiss-Prot Web Server, and the CD-HIT configuration was employed to remove redundant samples. Positive and negative samples for leukemia peptides were obtained from the UniProt Proteins web server database and investigated by Kim et al. The normalized dataset underwent a 20% reduction in homologous sequences, and the CD-HIT code was applied to the protein sequences [20].

### Data processing

**Fig. 2:**
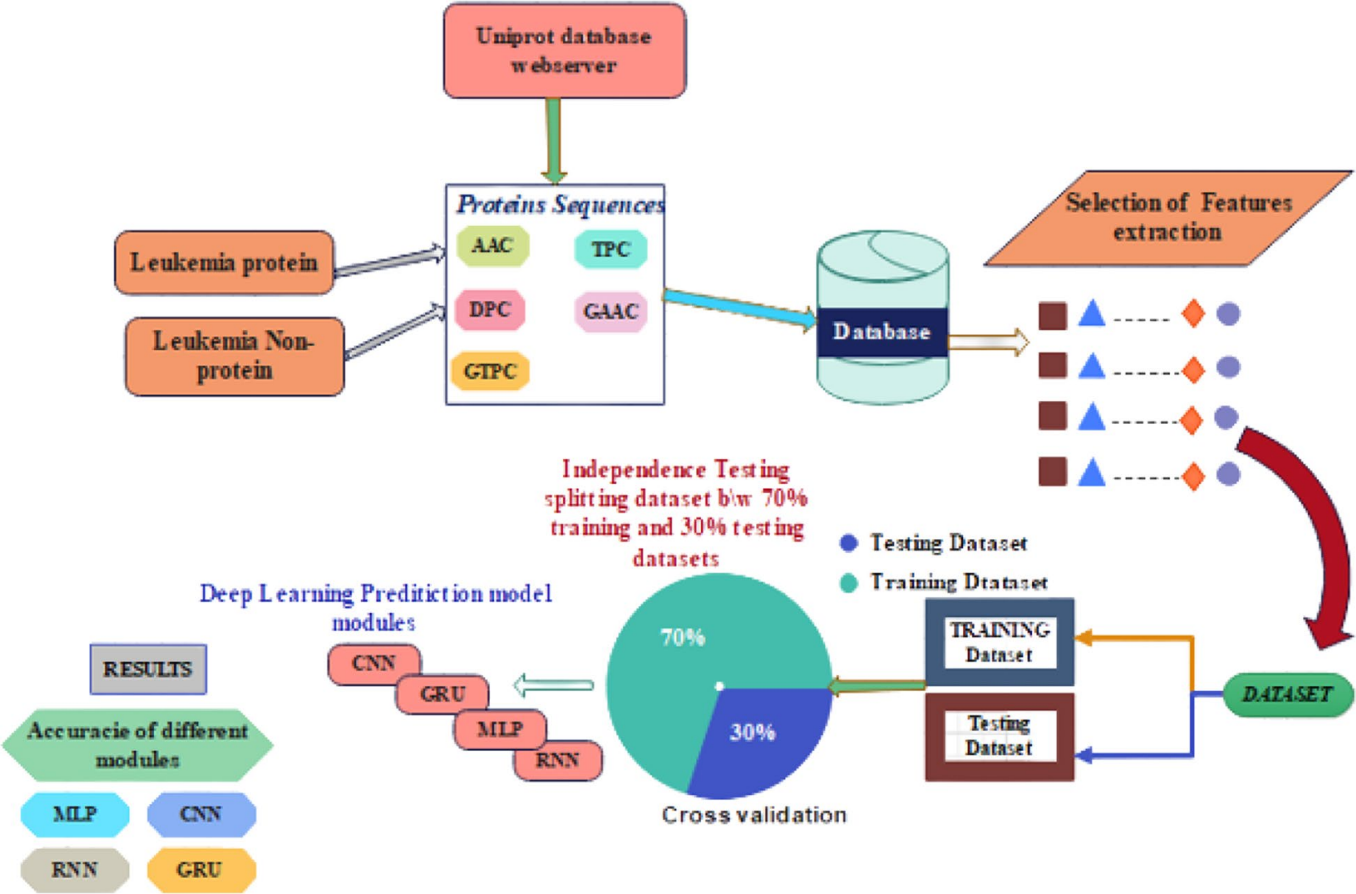
Architecture of the proposed approach

The accuracy of the results obtained in data analysis relies significantly on preprocessing, which includes the removal of noisy, inconsistent, missing, and irrelevant data. Noisy data pertains to incorrect entries in a dataset. Data preprocessing encompasses the removal of duplicates, and noisy data, as well as handling missing, inconsistent, and redundant information. Various tools, such as the Jalview tool [21] for sequence alignment and CD-HIT, can be employed to achieve this [20]. To cluster databases with high identification tolerance, ensuring precise and optimal results, the CD-HIT method is employed to minimize the occurrence of repeated peptides by removing sequences with more than 40% identical sequences, accounting for the overall protein sequence [20]. Extremely identical sequences [20]. The final training dataset comprised 897 leukemia amino acids and 973 non-leukemia polypeptides, while the independent test set included 256 leukemia peptides and 564 non-leukemia proteins. Deep learning theories were refined and examined for all assessed datasets, including a separate validation set with the model architecture structure presented in Fig. 2.

### Feature engineering method

For the selected leukemia datasets, the leukemia protein sequences are initially characterized through feature extraction by combining nine specific features. The aim is to construct an analog that can accurately identify leukemia proteins, utilizing the features extracted from polypeptides retrieved via the featured web server [22], with a focus on macromolecules.

#### Features investigation

This study focused on estimating sequence-based features of experimentally proven leukemia cancer peptides (LCP). Each biomolecule sequence was translated into a numerical vector based on the previously established attributes, with the aim of developing a deep learning model (Table 2).

i. **Amino Acid Composition (ACC):** represents the frequency of twenty different types of native amino compounds in certain peptide succession. When compared to the overall maximum number of characters in protein sequences, the 20 elements in a peptide sequence indicate the total quantity of occurrences of twenty different amino compositions [13, 22, 23].
ii. **Tripeptide Composition (TPC):** A cytokine is composed of many amino acids linked together by multiple polypeptides. Melanostatin belongs to several tripeptides that the human body produces (prolyl-leucyl-glycinamide). TPC represents an important potential source of inspiration for the composition of small molecule enhancers for living organisms. TPC is defined as Equation 2. $N_i$ indicates the number of the *i*th tripeptide. The TPC feature vector is composed of all possible tripeptides in a protein sequence, representing the arithmetical probability of those series of 3 amino acid compositions through tripeptide composition (TC). For a biological protein sequence of length L, the TPC feature vector has a length of 8000 (20 x 20 x 20). This vector, denoted as $d_1, d_2,...,d_{8000}$, transforms the protein sequence into an 8000-dimensional space [22, 24].
  1 $$\begin{aligned} f_i=\frac{w_i}{w-2} \end{aligned}$$
  2 $$\begin{aligned} f_{8000}=f_1,f_2,...,f_{8000}T \end{aligned}$$
iii. **Group of Tripeptide Amino Acid Composition (GTPC):** The tripeptide composition functionality provides information about the amino acid composition of three adjacent amino acids in a polypeptide sequence, enabling the inference of its functional properties. Using deep learning, it is possible to accurately predict the tripeptide composition functionality of a protein sequence, offering valuable insights into its biological activity and therapeutic potential. This approach has shown promise in identifying functionally important regions of proteins and can be applied in drug discovery and protein engineering. Overall, the identification of tripeptide composition functionality through deep learning can optimize the comprehension of protein combinations and aid in the invention of new treatments. The categorized tripeptide arrangement arises from the combination of TPC, GAAC, and GTPC [21, 22]. Table 3 displays the same aforementioned amino acid characteristics for the grouped tripeptide composition (GTPC) as well as the combo of TPC and GAA.
iv. **Group of Amino Acid Composition:** Another way to represent protein sequences in deep learning for classification and prediction tasks is through grouped GAACs. Genetic amino acid codes (GAACs) are developed by categorizing amino acids based on their physicochemical properties, recognizing that interactions between these categories compose a dynamic character in polypeptide anatomy and actions. The aliphatic group is one of five distinct groups. The first group, $g_1$, comprises aliphatic amino acids (GAVLMI), while the second group, $g_2$, includes aromatic amino acids (FYW). The remaining groups include the assured imputation group (KRH) as $g_3$, the negatively charged group (DE) as $g_4$, and the uncharged group (STCPNQ) as $g_5$ [22].
  3 $$\begin{aligned} (p)=\frac{N(p)}{N} p \in \{p_1,p_2,...,p_5\}c \end{aligned}$$
v. **Dipeptide Amino Acid Composition (DPC):** For labeling homologous (HUMAN) sequence data, Park and Kanehisa developed a novel sequential feature consisting of 400 characteristics that show the likelihood of each amino acid dipeptide. The suggested feature is made to counteract the quantity of dipeptides found in a particular homologous protease sequence. It is anticipated that sequence analysis will be more accurate and efficient when this characteristic is included in sequence labeling. This feature offers useful data on the likelihood of every amino acid dipeptide in a sequence, which is utilized to deduce the functional characteristics of the amino acid. All things considered, the suggested sequential characteristic might improve our knowledge of protein sequences and help with drug discovery and protein engineering [25]. To determine the function of the dipeptide amino acid composition in protein amino acid sequences, deep learning algorithms are used. Using a sizable dataset of known protein sequences with known dipeptide composition functionality, a deep neural network is trained using this technique. Subsequently, the dipeptide composition functionality of novel protein sequences may be highly accurately predicted using the trained model. This method can be applied to protein engineering and drug discovery since it has demonstrated promise in finding functionally significant areas of proteins. It is possible to deduce a protein sequence’s functional characteristics by using the dipeptide composition functionality, which offers details on the amino acid composition of nearby amino acids. Deep learning may be used in order to accurately forecast a protein sequence’s dipeptide composition functionality, which offers important insights into the biological activity and potential therapeutic applications of the protein [22, 26, 27].
  4 $$\begin{aligned} D_c(j,k)=\frac{N(j,k)}{N-1} \end{aligned}$$
  In this case, *N*(*j*, *k*) denotes the total quantity of occurrences the dipeptide (*j*, *k*) appears, while $D_c(j,k)$ denotes its frequency. There are *N* dipeptides in total in the protein sequence [22, 28].

**Table 2:** Types of features methods

Feature type	Description	Dimension frequency
AAC	Amino Acid Composition	20
DPC	Dipeptide Composition	400
TPC	Tripeptide Composition	8000
C\T\D	Composition\transition\distribution	147
GTPC	Group of Tripeptide composition	125
GAAC	Group of Amino Acid Composition	5

**Table 3:** For Amino acid and physiochemical compositions and its properties

Physiochemical properties	Amino Acid Compositions
Sequential	G, A, V, I, M
Aromatic	F, Y, W
Positive charge	K, R, H
Negative charge	D, E
Uncharged	S, T, C, P, N, Q

### Neural network architectures

Several deep learning architectures are adopted in this study. In deep neural networks, several architectures are proposed and utilized in the existing literature such as CNN, RNN, MLP, GRU, etc. [29]. Hybrid neural networks that combine these structures are also commonly used [13]. These complex networks have been successful in various fields [26]. In a recent study, researchers used Residual networks with 1-3 residual blocks and 2 fully connected layers, similar to models used for protein functional annotation. They applied Batch Normalization to all layers, a Weight dropout of 0.1 to the fully connected (FC) layers, and used the Adam optimizer with mean squared error loss function and different activation functions such as ReLU, tan(h), and sigmoid with uniform 50 weight initialization [13, 26, 28, 29]. The subsequent text provides a brief description of these architectures.

#### Convolutional neural network

CNN plays a vital role in the deep learning technique [30, 31]. A typical CNN architecture comprises convolutional, pooling, and fully connected layers. The convolutional layer gathers information from the input data using the convolution operation. Among the CNN layers, the 1D convolutional layer is the most commonly used, especially in analyzing protein sequences [30]. CNNs excel at capturing significant local features, making them widely applicable. For example, a convolution module can set up three layers with local connections and weight sharing to extract crucial local information [31]. Pooling layers serve to reduce the size of the parameter matrix and avoid overfitting by shrinking the spatial dimensions of the activation map. Adding these layers can also improve computational efficiency. CNNs frequently include dropout regularization techniques and ReLU activation functions in addition to the fundamental layers to induce nonlinearities and prevent overfitting during training [31].

For the CNN model, we used a ‘Batch_Size’ of 32 while the model was trained using 100 epochs. The CNN model contains flatten layers with 1D max-pooling and contains a total of 5 hidden layers containing 64 neurons and the dropout layers are used with a dropout rate of 0.1. Adam optimizer is used for optimization. Moreover, for filtering matrixes, we used ‘Filtter 1’, ‘Filter 2’, and ‘Filter 3’ with 32, 64, and 128 sizes with a kernel size of 3.

#### Recurrent neural network

When performing sequence labeling tasks for assessing the current input, RNNs are utilized because of their ability to compute sequences well. Long short-term memory (LSTM) neural networks and GRU neural networks are the two distinct subclasses of RNNs. Their purpose is to capture data in a sequential manner by applying “memory” and “forgetting” strategies that are predicated on past conditions.

The RNN model comprises 5 hidden layers each with 64 neurons and is trained using 100 epochs. It uses a ‘Batch_Size’ of 32, similar to other models used in this study. For optimization, the Adam optimizer is used and for loss data calculation we applied the binary_cross_entropy’ loss according to the label of the class.

#### Gate recurrent unit

GRU used in deep learning for processing sequential input, such as protein sequences, is the recurrent neural network design. GRU is a kind of RNN architecture used in deep learning. It has characteristics with the LSTM network, such as techniques for selectively controlling information flow. A single-member sequence (a vector with 100,000 dimensions) was processed by each record in the GRU model in 2014 to produce 32 GRU units. However, GRU is more computationally efficient than LSTM since it has fewer parameters. The gating mechanisms in the GRU network allow the recurrent unit to selectively recall or forget information from earlier time steps while also controlling the information flow within the unit. Its two gates the update gate and the reset shutter-manage the amount of newly contributed data to the current state and the amount of the prior concealed state that is kept. Long protein data sequences may be efficiently evaluated by the GRU network by selectively keeping or deleting information. Two dense layers of ten (10) and one (1) perceptron, respectively, are coupled with GRU outputs to provide predictions for a two-class issue. The first dense layer employs ReLU activation for predictions, whereas the classification layer utilizes sigmoid activation [32].

The GRU comprises 5 hidden layers, each having 64 neurons, and is trained using 100 epochs. For optimization, the Adam optimizer is used while 32 is used as the ‘Batch_Size’ for GRU. Furthermore, for data loss calculation, we applied ‘binary_cross_entropy’ loss according to the label of classes which is represented as class 0 and class 1. In the used model, we split the data in the ratio of 0.8 to 0.2 for training and testing, respectively which calculates the loss validation values through the validate function.

#### Multilayer perceptron

Deep learning employs multilayer perceptrons as modules for calculating hidden layers. Conventional neural networks use weights to achieve optimal outcomes, consisting of either a single layer or multiple layers of perceptrons. Predictions emerge through the output layer, also known as the visible layer when data is input into the input layer. There may be one or more hidden layers providing different levels of abstraction [32].

In multi-layer perceptron, we used a ‘Batch_Size’ of 32, and the model is trained using a total of 100 epochs. The Adam optimizer proved to be a good choice for optimizing the multilayer perceptron. A dropout rate of 0.1 is also used for the model. The ‘binary_cross_entropy’ is used as the loss parameter for the multilayer perceptron with respect to the label of the class.

### Evaluation metrics

A confusion matrix is the main evaluation metric from where all evaluation metrics are extracted. It contains four types of values true positive (TP), false positive (FP), true negative (TN), and false negative (FN). TP is the quantity of leukemia disease occurrences in protein sequences that are accurately predicted. Conversely, FP denotes the quantity of misclassified leukemia illnesses in protein sequences. Similarly, TN represents the number of successfully predicted non-leukemia diseases in protein sequences, whereas FN indicates the number of non-leukemia diseases that are wrongly predicted in protein sequences.

Seven indicators are used to evaluate the prediction model. The area under the receiver operating characteristic curve (AUC), the Matthews correlation coefficient (MCC), the sensitivity, specificity, accuracy, negative predictive value (negative-positive value), and precision are used. Moreover, the precision and recall of the model are combined using the F1 score, which is the harmonic mean of the accuracy and recall of the model [32].

Accuracy shows the efficiency of the model regarding correct predictions. Specificity shows the calculation of the efficacy of the model to predict negative samples [32]. Sensitivity demonstrates the possibility of forecasting positive examples for the model. Because it takes into account both classes despite unbalanced data, MCC is a stable measure. With both positive and negative samples, the model’s analytical capacity is explained by the MCC accuracy score [33]. The following formulas are used for these metrics.

5 $$\begin{aligned} Sensitivity = \frac{TP}{TP+FN} \end{aligned}$$

6 $$\begin{aligned} Specificity = \frac{TN}{TP+FP} \end{aligned}$$

7 $$\begin{aligned} Precision = \frac{TP}{TP+FP} \end{aligned}$$

8 $$\begin{aligned} Recall = \frac{TP}{TP+FN} \end{aligned}$$

9 $$\begin{aligned} F1 ~score = 2*\frac{Precision*Recall}{Precision+Recall} \end{aligned}$$

10 $$\begin{aligned} Accuracy = \frac{TP+TN}{TP+FP+TN+FN} \end{aligned}$$

11 $$\begin{aligned} MCC = \frac{TN*TP-FN*FP}{\sqrt{(FP+TP)(FN+TP)(FP+TN)(FN+TN)}}*100 \end{aligned}$$

## Results and discussion

To test the robustness of the predictor independent testing has been employed [34]. The first testing type is independence testing, where all the data has been used to test the model. As discussed in the previous section, we have experimented with multiple modules for deep learning, such as CNN, RNN, ANN, MLP, and GRU). The leukemia protein prediction model is really classified using binary classification, where the problem is divided into two categories: ‘0’ and ‘1’.

### Independent testing

This approach of splitting a dataset into a training set and a testing set is commonly employed in deep learning to assess the efficiency of models. The training set is utilized to train the model, while the testing set is used to evaluate its performance on new, unseen data. An application of this technique is in the analysis of leukemia peptide sequences. It enables the assessment of feature extraction effectiveness through the utilization of a deep learning model that employs protein sequence features. In order to evaluate the effectiveness of models, deep learning techniques frequently divide datasets into training and testing sets. The testing set is used to assess the model’s performance on fresh, untested data, whereas the training set is used to train it.

It is standard procedure to split a dataset into training and testing and 70% to 30% split is the most commonly used for machine learning. The precise ratios, however, might change based on the size, complexity, and needs of the given application, among other things. Peptide frequencies and molecular patterns are examples of characteristics found in protein sequences that are used to train and evaluate algorithms. The dataset is split into two distinct sets, one for testing and one for training, at random to carry out the split. It is crucial to make sure that in both sets, the percentage of samples belonging to distinct classes stays consistent.

The selected features are utilized to train the model or algorithm after the data has been set. Lastly, trials on fresh, untested data are used to evaluate the model’s performance. It is crucial to compare the predicted outputs of the model with the actual outcomes of analyzing the test data in order to assess the efficacy of feature extraction with deep learning modules. It is possible to calculate performance indicators like recall, accuracy, precision, and F1 score to evaluate how well this procedure is working. The model’s many components are tested independently using protein sequence characteristics to make sure they are resilient and able to handle new and untested data.

Table 4 shows the results for AAC and GAAC features. Results suggest superior performance of MLP and CNN models. According to the independence testing in the (AAC, GAAC) approach, it is determined that the MLP and CNN modules achieved the highest accuracy in identifying diseases in protein sequences. Both the CNN module and the MLP module showed 100% accuracy, precision, specificity, sensitivity, and Matthews’s correlation coefficient.

**Table 4:** Results of independency testing with amino acid composition and a group of amino acid composition

Model	Specificity	Sensitivity	MCC	Accuracy	Precision	Recall	F1 score
RNN	82.41	42.90	27.75	62.65	63.22	42.90	51.17
GRU	86.09	37.35	27.19	61.72	65.42	37.35	47.55
MLP	100	100	100	100	100	100	100
CNN	100	100	100	100	100	100	100

**Fig. 3:**
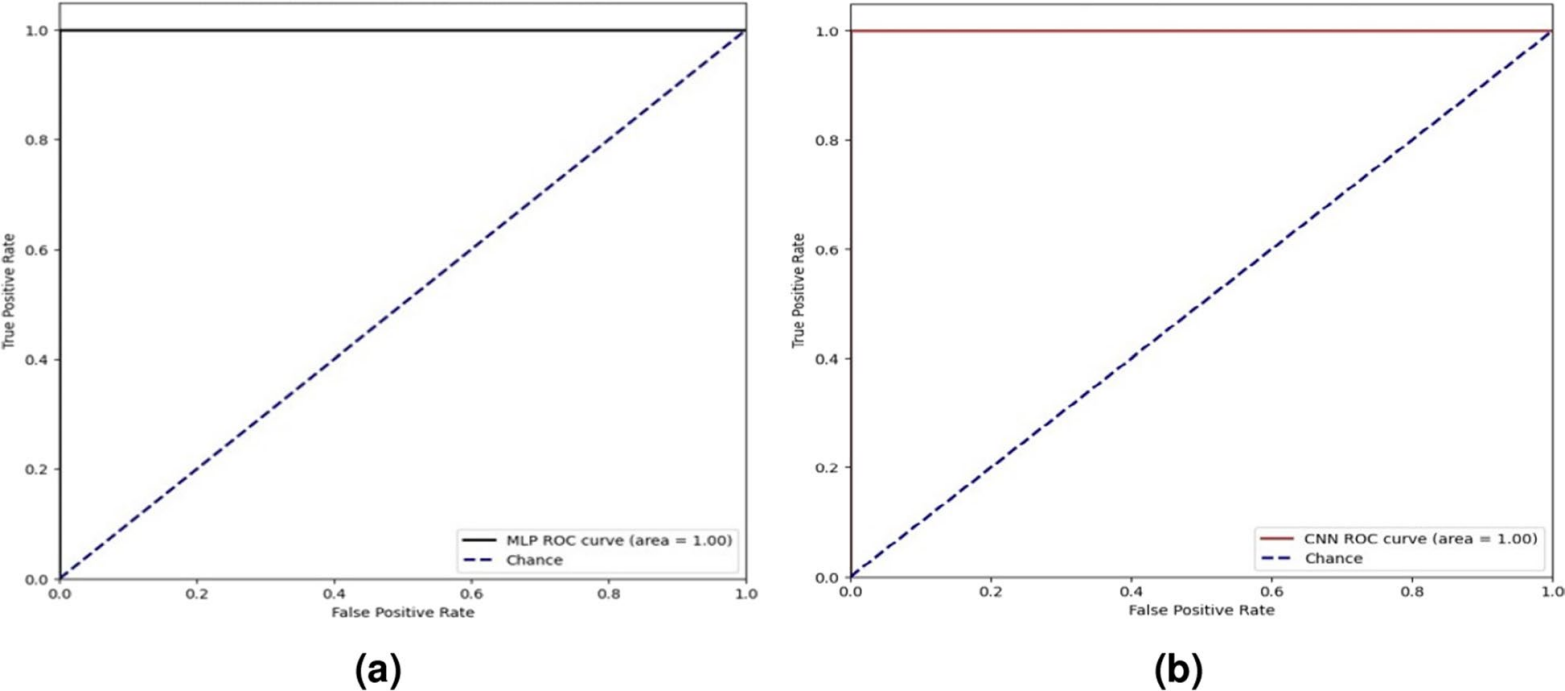
ROC graphs, **a** CNN modules for (AAC), and a group of (AAC, GAAC) composition, and **b** CNN modules for (ACC) and, a ( GAAC) composition

To construct a prediction model, biological features were integrated. In this study, independency testing for both the MLP and CNN modules demonstrated identical accuracy values of 100% for precision, recall, and F1-score when applied to testing datasets. ROC in Fig. 3, the area under the curve graph, shows 100% accuracy for MLP and CNN modules.

**Fig. 4:**
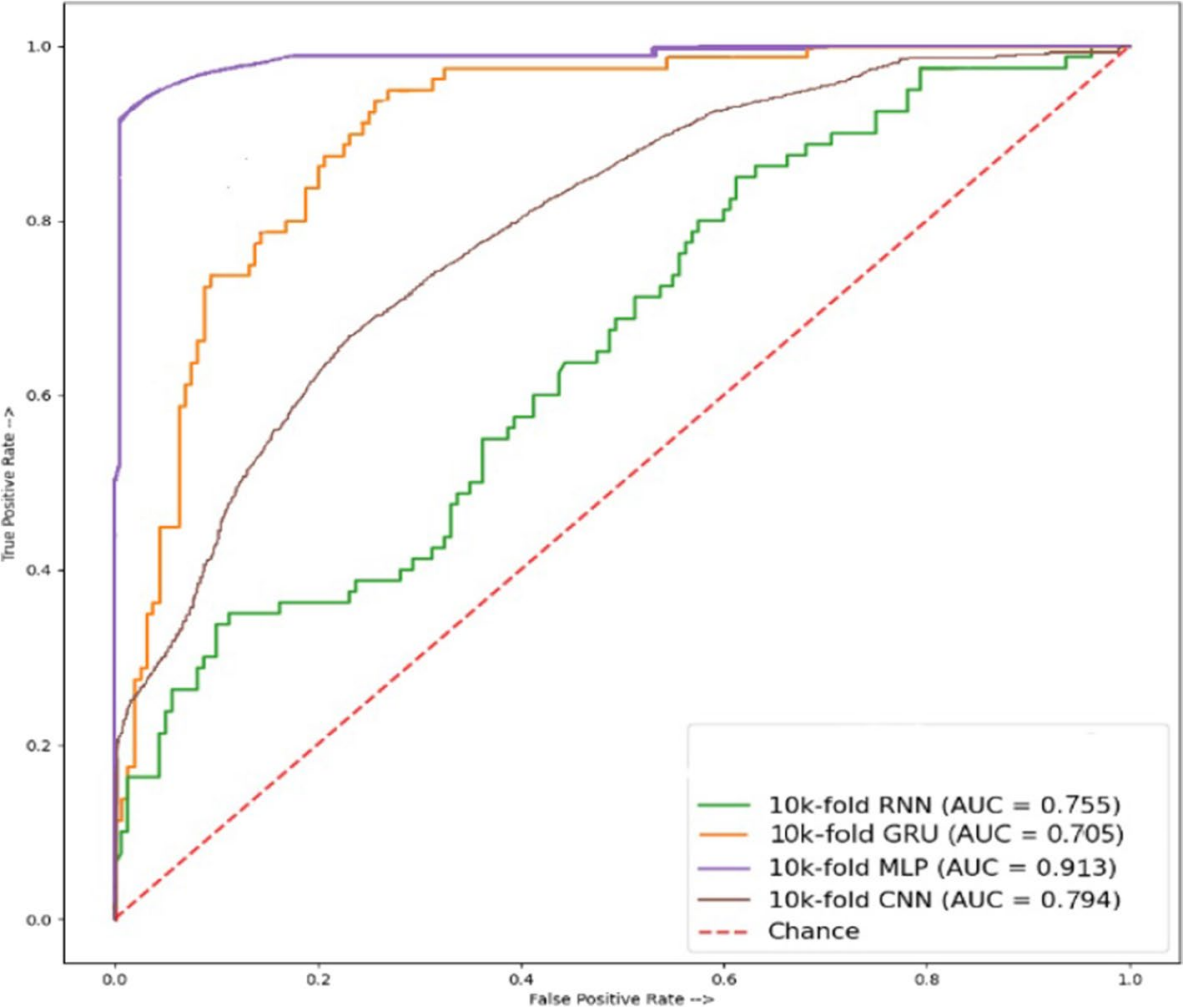
ROC accuracy of DPC peptide sequences cross-validation modules for amino acid composition

Results using the Dipeptide Composition (DPC) features are presented in Table 5, indicating the performance of rNN, GRU, MLP, and CNN deep learning models. These models are applied to determine their efficiency regarding the use of DPC features from the dataset for leukemia peptide detection. Results indicate a superior accuracy of 64.47% from GRU. It achieved an F1 score of 53.98%, specificity of 83.10%, sensitivity of 45.84%, recall of 45.84%, and precision of 65.85%, while the MCC is 31.45%. It is closely followed by the MLP model with a 64.11% accuracy. Results show degraded performance from these models when DPC is used for experiments. The area curve ROC graph shown in Fig. 4 for testing results indicates that MLP outperformed with a score of 0.913.

**Table 5:** Independence testing results of Dipeptide Composition (DPC)

Model	Specificity	Sensitivity	MCC	Accuracy	Precision	Recall	F1 score
RNN	82.06	41.27	25.71	61.67	61.85	41.27	49.51
GRU	83.10	45.84	31.45	64.47	65.85	45.84	53.98
MLP	75.86	0.5236	28.99	64.11	60.45	52.36	56.11
CNN	71.49	0.5497	26.69	63.23	0.5760	54.97	56.11

**Fig. 5:**
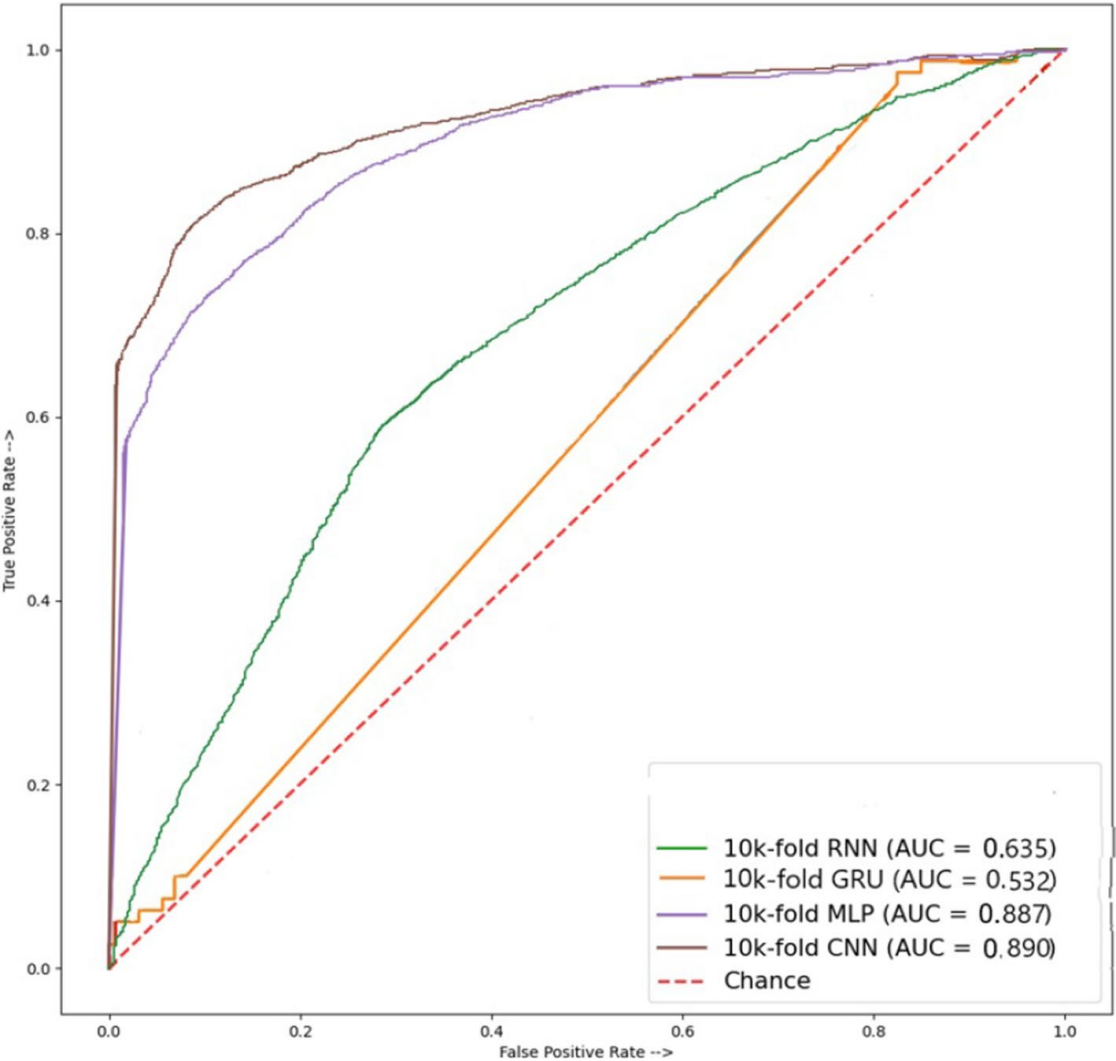
ROC accuracy of AAC, GAAC peptide sequences cross-validation modules for amino acid composition

Results for the jointly calculated independency testing (TPC, GTPC) are given in Table 6. TPC and GTPC features are used for this set of experiments to showcase the performance of deep learning models including CNN, RNN, MLP, and GRU. By applying these modules to testing data, we finalized the highest accuracy for disease identification. Experimental results suggest that MLP stands out with 80.00% accuracy, and specificity of 85.86%, sensitivity of 82.36%, recall of 76.36%, MCC of 88.99%, precision of 70.45%, and F1 score of 86.11%. RNN, GRU, and CNN have substantially lower scores for these metrics when TPC and GTPC are used for experiments. The ROC graph shown in Fig. 5 for testing results indicates that CNN performed outstandingly with a score of 0.890.

**Table 6:** Results of independence testing of Tripeptide Composition and a group of (TPC, GTPC) compositions

Model	Specificity	Sensitivity	MCC	Accuracy	Precision	Recall	F1 score
RNN	75.86	49.75	17.77	62.80	59.22	49.75	54.07
GRU	80.22	41.59	23.73	60.91	59.71	41.59	49.03
MLP	85.86	82.36	88.99	80.00	70.45	76.36	86.11
CNN	71.49	54.97	26.69	63.23	57.60	54.97	56.11

### Validation using 10-fold cross-validation and independent dataset testing

Despite the results obtained using independence testing, the models might not perform well on unseen data indicating the model’s overfitting to a particular class of dataset [22, 31]. Even if a large dataset is available, it might not be enough to evaluate the prediction model’s accuracy. K-fold cross-validation is a good solution for this problem [34]. Independent testing is quite detailed and can yield varying outcomes for a given benchmark. In situations when there is no clear record to support the model’s predictions, cross-validation is the most effective method for verifying and making sure a model is operating as intended [32].

When splitting a record into k single folds for cross-validation, k is the number of folds indicating how many portions of the dataset are made [26]. Each run chooses a different data % at random to verify the remaining data, ensuring that every fold of the data is utilized for both training and testing [21, 22]. The average of all accuracy values is the outcome. The dataset’s positive and negative examples were obtained using an identical number of records [22, 27]. After choosing a set of random values, k = 10 subgroups were created. For a variety of real-world data, cross-validation performs better than other techniques. These techniques are applied to random or segmented data selection for testing [21, 22, 31]. A substantially 10-fold technique for cross-validation was used to produce the results displayed in Tables 7.

**Table 7:** Results of cross-validation using Amino acid composition and a group of (AAC, GAAC) compositions

Model	Specificity	Sensitivity	MCC	Accuracy	Precision	Recall	F1 score
RNN	96.25	98.75	93.63	97.08	92.94	98.75	95.75
GRU	95.62	92.5	87.85	94.58	91.35	92.50	91.92
MLP	98.12	95.00	93.42	97.83	96.20	95.00	95.59
CNN	97.50	100	96.36	98.33	95.23	100	95.59

**Fig. 6:**
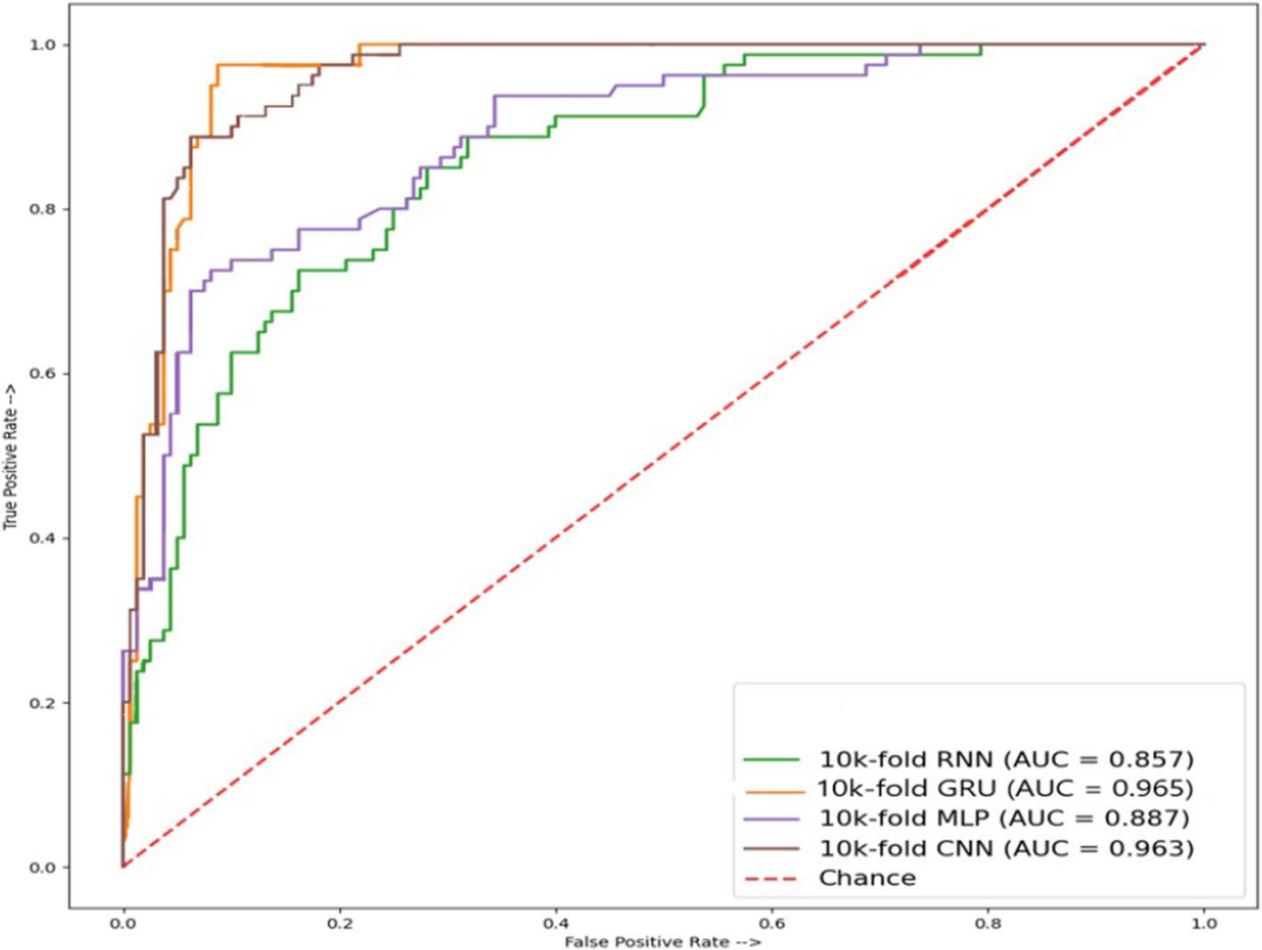
ROC accuracy of AAC and GAAC combined peptide sequences result of cross-validation test

According to cross-validation testing for AAC, and GAAC composition results given in Table 7, the CNN model outperforms other models. It achieves an accuracy of 98.33%, specificity of 97.50%, sensitivity of 100%, MCC of 96.36%, and precision of 95.23% while the recall and F1 scores are 100%, and 92.00%, respectively. Figure 6 shows that GRU shows better performance regarding the ROC curve with a score of 0.965.

**Fig. 7:**
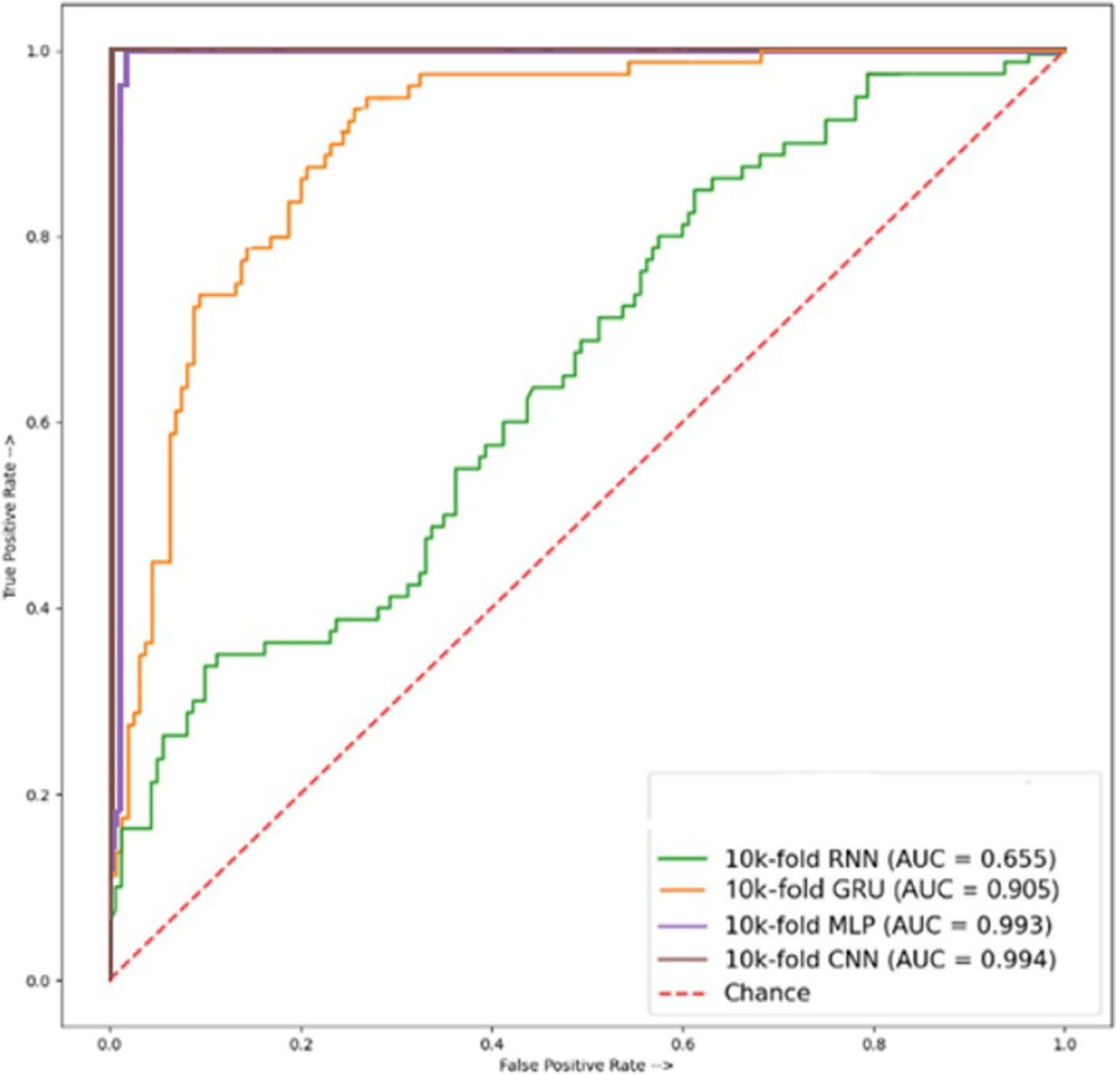
ROC Accuracy of (TPC, GTPC) combined result of peptide sequences cross-validation test

Cross-validation results for TPC, and GTPC composition using CNN, RNN, MLP, and GRU, are given in Table 8. The overall highest performance is achieved using the CNN model. It obtains an accuracy of 96.69%, specificity of 95.50%, sensitivity of 100%, MCC of 62.93%, precision of 97.23%, recall of 100%, and F1 scores of 97.59%. The best performance concerning the area under the curve ROC is obtained by the CNN model as well, as illustrated in Fig. 7, with a 0.994 score.

**Table 8:** Results of cross-validation using TPC and a group of (GTCP) peptide sequences

Model	Specificity	Sensitivity	MCC	Accuracy	Precision	Recall	F1 score
RNN	100	17.06	10.21	66.66	19.04	17.06	31.08
GRU	98.75	05.00	11.32	67.50	91.35	50.00	09.30
MLP	94.37	100	93.10	96.23	96.20	95.00	95.59
CNN	95.50	100	62.93	96.69	97.23	100	97.59

**Fig. 8:**
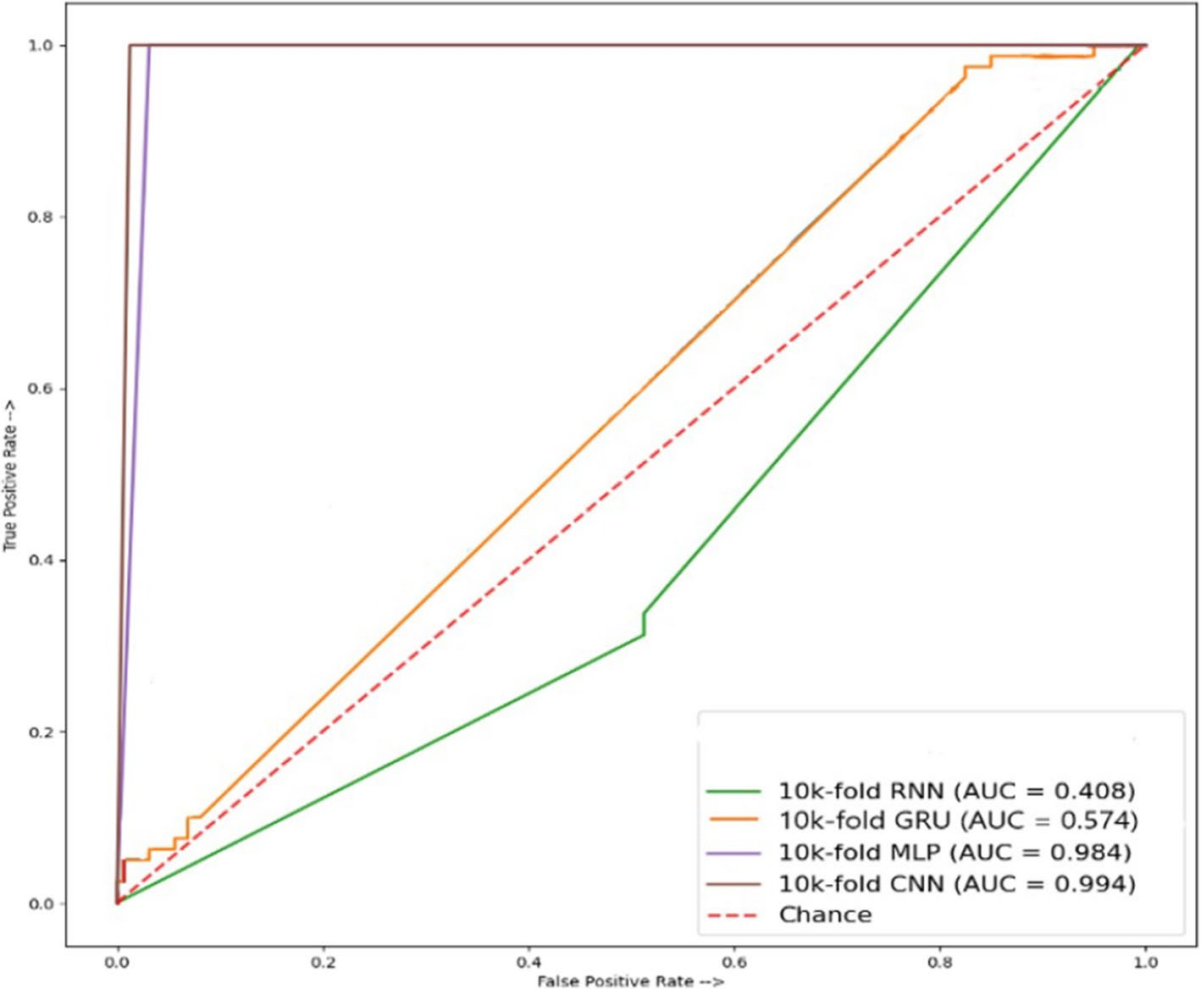
ROC accuracy of combined (TPC, GTCP) peptide sequences cross-validation modules for amino acid composition

Along the same direction, cross-validation for deep learning models including CNN, RNN, MLP, and GRU is carried out using the TPC and GTPC composition and results are displayed in Table 9. Results indicate superior performance of CNN model when using TPC, and GTPC composition. An accuracy of 98.33% is obtained by the CNN model. It also obtains better results regarding other evaluation metrics including a specificity of 95.00%, sensitivity of 100%, and MCC of 92.93%. The area under the curve ROC, as shown in Fig. 8 shows a score of 0.994 for CNN which is the best among all the models.

**Table 9:** Results of cross-validation using Dipeptide Composition (DPC)

Model	Specificity	Sensitivity	MCC	Accuracy	Precision	Recall	F1 score
RNN	96.25	98.75	93.63	97.08	92.94	98.75	95.75
GRU	95.62	92.5	87.85	94.58	91.35	92.5	91.92
MLP	98.12	95.00	93.421	97.83	96.20	95.00	95.59
CNN	97.50	100	76.33	98.33	95.23	100	95.59

### Discussion

Determining leukemia proteins using biological characteristics can be a time-consuming and difficult task, necessitating the use of computer-aided diagnosing methods. These technologies are critical for speeding up and simplifying the discovery of leukemia proteins. While these proteins have the potential to cause disorders in the human body, they are also important in the development of therapies to treat drug addiction. Furthermore, leukemia proteins play a crucial function in the creation of biomedicine and advancements in the field of life sciences.

Multiple deep-learning models including CNN, RNN, MLP, and GRU, are used to identify leukemia illnesses in protein sequences. The independency testing examination of these modules in this work demonstrates that MLP and CNN consistently have the highest accuracy in AAC, and GAAC compositions, reaching 100% in all calculations for detecting leukemia illnesses in protein sequences. TPC and GTPC combined calculation results showed good results, and TPC, and GTPC together calculated the results for DPC.

Cross-validation experiments excel at detecting detailed patterns within large datasets, even when presented with a large number of characteristics and samples. Only a subset of the leukemia dataset’s thousands of features was used for the deep learning model with their groups combined peptide order and individual peptide sequences for efficient analysis, using some state-of-the-art techniques to estimate its productiveness. Deep learning is currently at the forefront of machine learning methodologies, generating excellent results in various fields such as healthcare, medicine, and bioinformatics. The usefulness of deep learning approaches has been rigorously proven, cementing their place as the most recent developments in this domain [32]. Deep learning has also shown promising results in several medical domains, including the diagnosis of leukemia diseases by converting protein sequence vectors into binary form with ‘0’ and ‘1’.

## Conclusion

This study analyzes the use of various protein compositions for leukemia detection employing multiple deep-learning models including CNN, RNN, MLP, and GRU. With the combined paired peptide order and individual peptide sequences for analysis, a more in-depth and accurate detection of disease appearances in the human body through proteins, using some state-of-the-art techniques, was employed to estimate its productivity. Results suggest that CNN outperforms other models in properly identifying leukemia from protein sequences. A thorough independent testing examination of these modules in this work demonstrates that mostly MLP, CNN, and GRU consistently have the highest accuracy in all peptide combinations of sequences. AAC and GAAC accuracy of 100% shows the highest in modules like MLP and CNN, and their ROC graph shows 100% results in MLP and CNN modules, while TPC and GTPC show an accuracy of 80.00% shows using the MLP model, and its ROC graph of 0.890 shows the highest results using the CNN. DPC shows an accuracy of 64.47% with GRU and its ROC graph of 0.913 shows the highest results when using MLP. In cross-validation testing, AAC, and GAAC peptide sequences accuracy of 98.33% shows the highest accuracy using CNN, while the best ROC score of 0.965 is obtained using GRU. For TPC and GTPC, an accuracy of 96.69% is achieved using the CNN model which also obtains the best ROC score of 0.965. DPC composition shows a cross-validation accuracy of 98.33% using the CNN which also shows the best ROC score of 0.994. Deep learning models show the potential of automated leukemia detection using various protein compositions and can further be investigated to obtain better results in the future.

## Data Availability

The datasets and implementation code for this study can be found on GitHub using the following link https://github.com/SeherKhawaja/Leukemia-dieases.
