# Correction: Prediction of leukemia peptides using convolutional neural network and protein compositions

**DOI:** 10.1186/s12885-024-12756-y

**Published:** 2024-08-08

**Authors:** Seher Ansar Khawaja, Muhammad Shoaib Farooq, Kashif Ishaq, Najah Alsubaie, Hanen Karamti, Elizabeth Caro Montero, Eduardo Silva Alvarado, Imran Ashraf

**Affiliations:** 1 School of System and Technology, University of Management and Technology, Lahore, 54000 Pakistan; 2 Department of Computer Sciences, College of Computer and Information Sciences, Princess Nourah bint Abdulrahman University, P.O. Box 84428, Riyadh, 11671 Saudi Arabia; 3 Universidad Europea del Atlántico, Isabel Torres 21, Santander, 39011 Spain; 4 Universidad Internacional Iberoamericana Arecibo, Puerto Rico, 00613 USA; 5 Universidade Internacional do Cuanza, Cuito, Bié, Angola; 6 Universidad Internacional Iberoamericana, Campeche, 24560 México; 7 Universidad de La Romana, La Romana, Dominican Republic; 8 República Dominicana 8 Information and Communication Engineering, Yeungnam University, Gyeongsan, 38541 Korea

**Correction: BMC Cancer 24, 900 (2024)**

10.1186/s12885-024-12609-8

Following publication of the original article [1], the authors identified an error in the author name of Seher Ansar Khawaja.

The incorrect author name is: Sehar Ansar Khawaja

The correct author name is: Seher Ansar Khawaja

The author group has been updated above and the original article [1] has been corrected.

## References

[1] Khawaja SA, Farooq MS, Ishaq K, et al. Prediction of leukemia peptides using convolutional neural network and protein compositions. BMC Cancer. 2024;24:900. 10.1186/s12885-024-12609-8. 39060972 10.1186/s12885-024-12609-8 PMC11282659

